# Building a Simple Model to Assess the Impact of Case Investigation and Contact Tracing for Sexually Transmitted Diseases: Lessons From COVID-19

**DOI:** 10.1016/j.focus.2023.100147

**Published:** 2023-09-25

**Authors:** François M. Castonguay, Harrell W. Chesson, Seonghye Jeon, Gabriel Rainisch, Leah S. Fischer, Biswha B. Adhikari, Emily B. Kahn, Bradford Greening, Thomas L. Gift, Martin I. Meltzer

**Affiliations:** 1 Health Economics and Modeling Unit, Division of Preparedness and Emerging Infections, National Center for Emerging and Zoonotic Infectious Diseases, Centers for Disease Control and Prevention, Atlanta, Georgia; 2 Department of Health Management, Evaluation and Policy, School of Public Health, University of Montréal, Montréal, Québec, Canada; 3 Centre for Public Health Research (CReSP), Montréal, Québec, Canada; 4 National Center for HIV, Viral Hepatitis, STD, and TB Prevention, Division of STD Prevention, Centers for Disease Control and Prevention, Atlanta, Georgia

**Keywords:** Sexually transmitted diseases, case investigation, contact tracing and partner notification, cases averted, modeling

## Abstract

**Introduction:**

During the COVID-19 pandemic, the U.S. Centers for Disease Control and Prevention developed a simple spreadsheet-based tool to help state and local public health officials assess the performance and impact of COVID-19 case investigation and contact tracing in their jurisdiction. The applicability and feasibility of building such a tool for sexually transmitted diseases were assessed.

**Methods:**

The key epidemiologic differences between sexually transmitted diseases and respiratory diseases (e.g., mixing patterns, incubation period, duration of infection, and the availability of treatment) were identified, and their implications for modeling case investigation and contact tracing impact with a simple spreadsheet tool were remarked on. Existing features of the COVID-19 tool that are applicable for evaluating the impact of case investigation and contact tracing for sexually transmitted diseases were also identified.

**Results:**

Our findings offer recommendations for the future development of a spreadsheet-based modeling tool for evaluating the impact of sexually transmitted disease case investigation and contact tracing efforts. Generally, we advocate for simplifying sexually transmitted disease–specific complexities and performing sensitivity analyses to assess uncertainty. The authors also acknowledge that more complex modeling approaches might be required but note that it is possible that a sexually transmitted disease case investigation and contact tracing tool could incorporate features from more complex models while maintaining a user-friendly interface.

**Conclusions:**

A sexually transmitted disease case investigation and contact tracing tool could benefit from the incorporation of key features of the COVID-19 model, namely its user-friendly interface. The inherent differences between sexually transmitted diseases and respiratory viruses should not be seen as a limitation to the development of such tool.

## HIGHLIGHTS

• Lessons learned from the COVID-19 pandemic can help better manage other diseases.
• Tools developed to estimate the impact of policies can be adapted to other diseases.
• Differences across diseases should not be seen as a limitation for tool development.
• Simplicity of the user interface is key for tool adoption by public health practitioners.

## INTRODUCTION

Case investigation and contact tracing (CICT) consists of identifying, contacting, and managing potential next-generation cases. These are individuals (hereafter referred to as contacts) who may have been exposed to some pathogen because of their proximity to or relationship with an infected individual.^1^ The primary goal of CICT is to interrupt the chain of transmission.

The U.S. Centers for Disease Control and Prevention (CDC) built COVIDTracer and COVIDTracer Advanced modeling tools to help assess the impact of coronavirus disease 2019 (COVID-19) CICT program^2^ (see CDC's website https://www.cdc.gov/coronavirus/2019-ncov/php/contact-tracing/COVIDTracerTools.html to access the tools and accompanying manuals. A special edition of the tool is available at https://www.cdc.gov/ncezid/dpei/resources/covid-tracer-Advanced-Special-edition.xlsm, which is a modification of the first version of the tool because it enables users to assess the impact of CICT in terms of cases and hospitalizations averted; see online supplementary content of the study by Rainisch et al.^3^ for a user manual of this version of the tool). The tool permits users to estimate the impacts of CICT versus other nonpharmaceutical interventions (NPIs) such as facemask wearing, restrictions on large gatherings, and school and business closures. Using these tools, researchers found that 4%–97% of remaining cases (not prevented by other NPIs) were prevented by CICT across 14 jurisdictions in the U.S. during the summer of 2020.^4^ Another study using similar methods but with an expanded set of data from 23 jurisdictions covering nearly half of the U.S. population estimated that CICT prevented 1.11 million COVID-19 cases and 27,231 hospitalizations during a 60-day period spanning from mid-November 2020 to mid-January 2021.^3^ Other published^5^ and ongoing^6^, ^7^ work also uses this tool.

This paper describes CDC's COVIDTracer spreadsheet-based tool, the design criteria, and both the disease- and jurisdiction-specific data requirements that users must enter to produce results that are of practical value for practicing public health officials. We then identify some key challenges and potential solutions to producing similar tools to assess the impact of CICT activities on 3 sexually transmitted diseases (STDs): chlamydia, gonorrhea, and syphilis.

## METHODS

The epidemiologic parameters and structure of the COVIDTracer modeling tool and how they were used to estimate the impact of COVID-19 CICT were reviewed. In addition, the existing spreadsheet-based tools that assess the impact of STD CICT programs were reviewed. Discussions were held among the authors to assess whether the methodology used by COVIDTracer could be readily adapted to STDs. Potential challenges that might arise when developing such a tool as well as possible ways to address these challenges were also assessed. Existing features of the COVID-19 tool that could be most useful for evaluating the impact of CICT on STDs were identified. These discussions focused on the key epidemiologic differences between STDs and respiratory diseases, such as mixing patterns, incubation period, duration of infection, the availability of treatment, and the unit of time appropriate for tracking different diseases.

The review of the COVIDTracer tool and its application was based on publicly available data^2^ as well as the authors’ expertise and experience with the model. Similarly, the review of existing spreadsheet-based tools for estimating the benefits of CICT for STDs relied on publicly available data^8^^,^^9^ as well as the authors’ expertise and experience with the models.

## RESULTS

### Building a Simple Interactive Modeling Tool for COVID-19 Contact Tracing

**Figure 1:**
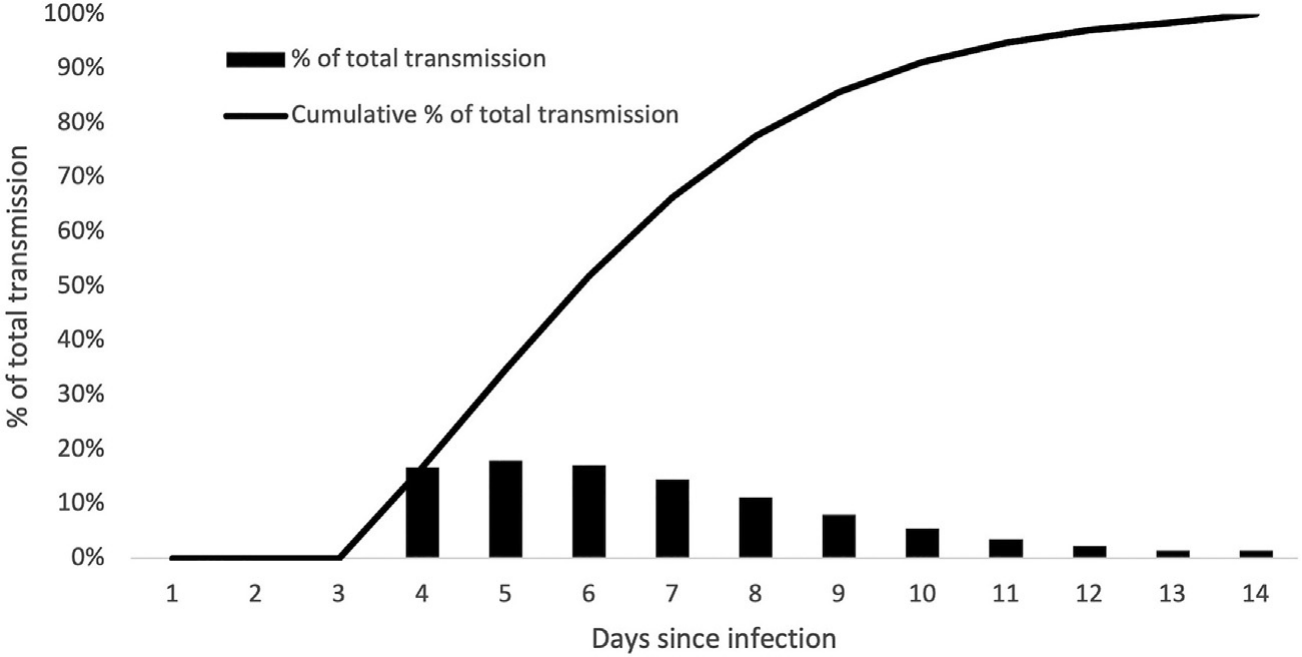
Estimated risk of infecting others each day since COVID-19 infection.

*Notes:* Bars represent the daily risk, and the line represents the cumulative percentage of total transmission. Sources: He et al.^13^ and Ferretti et al.^14^

CDC's COVIDTracer uses a combination of (1) epidemiologic data from the scientific literature and jurisdiction-specific data related to (2) the spread of severe acute respiratory syndrome coronavirus 2 (SARS-CoV-2), the virus that causes COVID-19; (3) COVID-19 vaccination levels; (4) demographic data such as population size and age distribution; and (5) CICT performance. Epidemiologic parameters and initial values used in COVIDTracer are presented in Table 1.^10^, ^11^, ^12^, ^13^, ^14^ Infectivity in COVIDTracer is based on 4 epidemiologic parameters. The first is the basic reproduction number (R_0_), which is the average number of infections caused by an infected individual in a population that has no immunity or protection against infection (i.e., all members are entirely susceptible).^15^ The effective reproduction value is recalculated each day in the model and accounts for the effect of any interventions the user wishes to include. The other 3 epidemiologic parameters are the length of time between exposure to the virus and the onset of infectiousness (i.e., the latent period), the duration of infectiousness, and the amount of viral shedding over that infectiousness period because the risk of infecting others varies over the illness course. Figure 1 shows the daily infectivity of COVID-19.

**Table 1:** List of Epidemiologic Parameters Used in the COVIDTracer Tool

Parameter	Default value	Source
New infections per case (R_0_)	2.5–5.0	Alimohamadi et al.^10^ and Liu and Rocklov^11^
Infected but not yet infectious period^a^	2–3 days	Xiang et al.^12^
Presymptomatic and contagious (infectious) period	2 days	He et al.^13^ and Ferretti et al.^14^
Symptomatic and contagious (infectious) period	9–10 days	He et al.^13^ and Ferretti et al.^14^
Daily infectivity distribution^b^	Figure 1	He et al.^13^ and Ferretti et al.^14^
Percentage of asymptomatic cases	40%	CDC COVID-19 pandemic planning scenarios^c^
Infectiousness of asymptomatic cases (relative to that of symptomatic cases)	75%	CDC COVID-19 pandemic planning scenarios^d^

a Latent period.

b Intensity of infectivity over the entire duration of illness.

c See CDC's website: https://www.cdc.gov/coronavirus/2019-ncov/hcp/planning-scenarios.html.

d See CDC's website https://www.cdc.gov/coronavirus/2019-ncov/php/contact-tracing/COVIDTracerTools.html to access the tools and accompanying manuals. A special edition of the tool is available at https://www.cdc.gov/ncezid/dpei/resources/covid-tracer-Advanced-Special-edition.xlsm, which is a modification of the first version of the tool because it enables users to assess the impact of CICT in terms of cases and hospitalizations averted; see online supplementary content of the study by Rainisch et al.^3^ for a user manual of this version of the tool. CDC, Centers for Disease Control and Prevention.

Users also need to provide the cumulative number of cases as of the start date of the desired analysis period, the number of cases over the immediate past 14 days, the cumulative number of people vaccinated (if any), and the total population in the jurisdiction. These data are used to model the disease trajectory, providing a picture of the size and speed of the spread of COVID-19. The user may also enter the risk of hospitalization by age group to produce an estimate of age group–specific hospitalizations averted.

Finally, a user enters jurisdiction-specific data about their CICT program's performance: the percentage of cases and contacts who were effectively isolated and quarantined owing to CICT efforts and the number of days (from infection) needed to do so. In the studies of CICT impact mentioned earlier,^3^^,^^4^ these entries were calculated from a broader set of performance metrics and assumptions summarizing community receptivity to CICT and program logistics.

### Using COVIDTracer to Estimate the Impact of COVID-19 Case Investigation and Contact Tracing

**Figure 2:**
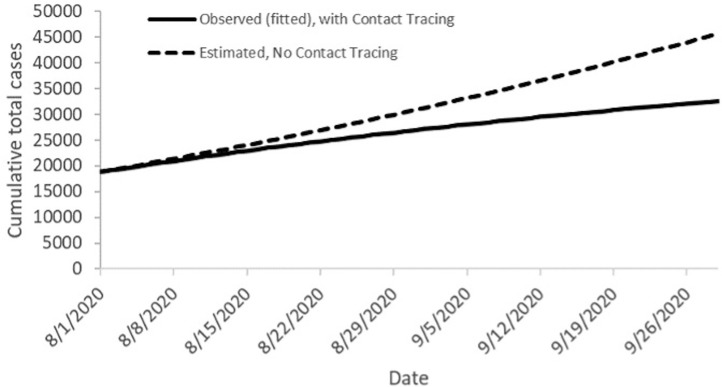
Epidemic curves produced by COVIDTracer with and without CICT programs, illustrating what might have occurred had the programs not been implemented.

*Notes:* The solid line is the observed cumulative case count with both CICT and other NPIs implemented. The dashed line is the model-generated curve illustrating an estimate of cumulative cases that might have occurred if CICT had not been implemented and only other NPIs were implemented. The differences between the solid and dashed lines show the benefits of CICT, with a greater divergence between the 2 indicating a greater impact. These results assume that the effects of CICT and other NPIs were constant over the 60 days shown. CICT, case investigation and contact tracing; NPI, nonpharmaceutical intervention.

Users can calculate CICT effectiveness using field-based data and can estimate the reductions in transmission due to other NPIs by fitting the curve of cases estimated by COVIDTracer to the plot of the jurisdiction's reported cases over time. To obtain a good fit (i.e., small deviation between the 2 curves, measured by the mean squared error), the user adjusts the percentage reduction in transmission attributed to NPIs until the model-generated curve of cumulative cases closely matches the reported cases. Then, to estimate the impact of CICT, the user sets the CICT effectiveness to zero while maintaining the estimated effectiveness of other NPIs constant. This produces estimates of what would have happened in the absence of CICT (the dashed line in Figure 2).

One advantage of using a spreadsheet-based tool such as COVIDTracer is that it becomes relatively easy to perform sensitivity analyses. For instance, Jeon et al.^4^ used COVIDTracer to identify the improvement in cases averted associated with 1-day incremental improvements in contact notification. Such sensitivity analyses help policymakers to identify the most impactful programmatic changes and expected impacts from such changes.

### Existing Tools to Assess the Impact of Sexually Transmitted Disease Prevention Activities

Two spreadsheet-based tools are available to STD programs that can provide estimates of the value of CICT. One tool is STIC (Sexually Transmitted Infection Costs) Figure,^8^^,^^16^ a spreadsheet-based program that allows users to estimate the costs averted by their STD program activities. These activities include treatment of people with gonorrhea, chlamydia, or syphilis, whether identified through CICT or not. STIC Figure also calculates the benefits of treating partners presumptively by applying assumptions about the percentage of partners who are themselves infected. In STIC Figure, the reduced onward transmission of STDs is estimated by assuming that 0.5 new infections are prevented for each infected partner treated.

The other tool is SPACE (STD Prevention Allocation Consequence Estimator) Monkey,^9^ which allows users to estimate the impact of budget cuts (or budget increases) on their program in terms of the change in the number of STD cases and the associated cost. SPACE Monkey generates these estimates using 2 approaches.^17^ The first approach applies estimates from published regression analyses of the association between STD prevention funding and subsequent STD rates at the state level. The second approach allows users to assume that the entirety of the budget cut (or increase) is focused on funding for CICT. SPACE Monkey estimates the impact of changes in funding for CICT on the basis of an analysis of historical records of reported gonorrhea cases and partner notification services in New York State, which estimated that each 10% increase in CICT activities was found to reduce gonorrhea rates by approximately 2%.^18^

Although STIC Figure and SPACE Monkey allow STD programs to calculate some of the benefits of CICT, these tools offer a more limited set of CICT-specific output than available through COVIDTracer. For example, they cannot be used to estimate the benefits of increasing notification speed or the percentage of cases interviewed or contacts notified. STIC Figure and SPACE Monkey also rely on relatively simple assumptions based on limited data when estimating the benefits of CICT. The appropriateness of these assumptions likely varies across jurisdictions depending on various epidemiologic, demographic, and programmatic characteristics.

### Challenge 1: Accounting for the Impact of Sexually Transmitted Disease Diagnosis and Treatment

STD diagnosis can play a substantial role in mitigating disease transmission because sexual behavior changes may occur after a positive diagnosis, which makes it difficult to distinguish between the impact of CICT and isolation and quarantine that would have occurred irrespective of individuals having contact with their health department (e.g., motivated by guidance from a treating physician or because of the increasing role of self-disclosure at preventing transmission^19^). This is not necessarily a limitation of simple tools because Rainisch et al.^3^ used COVIDTracer to address this potential through a sensitivity analysis. However, COVIDTracer does not consider the treatment of infected or exposed individuals to SARS-CoV-2 because there is no postexposure prophylactic treatment for COVID-19 to prevent further transmissions. In contrast, contacts of people with STDs such as syphilis, gonorrhea, and chlamydia can be epidemiologically treated for their potential infections. These treatments have the added benefit of potentially averting subsequent infections in the population. In addition, the treatment of gonorrhea and chlamydia in women identified through CICT can prevent long-term sequelae such as pelvic inflammatory disease and infertility.^16^^,^^20^ Ideally, tools that quantify the benefits of CICT for STDs would include not only the benefits in terms of the number of future STD cases averted but also the benefits of preventing long-term sequelae among infected partners who are identified and treated through CICT.

#### Addressing Challenge 1

To account for the treatment of infected individuals, an STD tool would need to account for the proportion of infected individuals treated and when treatment was initiated. These data could be used to stratify the risk of transmission posed by treated and untreated individuals and, over time, for treated individuals. The latter may be used to further quantify the impact of CICT programs if treatment is initiated earlier because of interactions between cases and contacts and their health departments.

### Challenge 2: Fitting a Model-Generated Plot of Cumulative Cases to the Plot of Reported Sexually Transmitted Disease Cases

Users of COVIDTracer estimate reductions in transmission due to NPIs other than CICT by fitting the model-generated epidemic curve to the plot of the reported cases—the fitting process for STDs would account for other NPIs, such as condom use, partner selection, or pretesting before sexual contact. However, fitting a model-generated epidemic curve to the plot of reported chlamydia, gonorrhea, or syphilis cases might be difficult for other reasons. For example, the epidemic curves for these STDs can fluctuate from one disease to another and over time, requiring a variety of robust fitting methods. Implementing such statistical flexibility into a spreadsheet-based tool may prove challenging, even for a tool that is STD specific. For example, fluctuations in case counts may depend upon the unit of time (i.e., daily, weekly, or monthly) appropriate for tracking an STD. In addition, the degree of asymptomatic infections leading to under-reported and underdiagnosed cases over time may also vary from one STD to another, but this issue is not unique to STDs and applies also to COVID-19. Accounting for such in the fitting process may also contribute to larger uncertainties.

#### Addressing Challenge 2

To allow for variations in disease transmission over time and account for the appropriate unit of time at which these STDs are being tracked, the model-fitting process used in COVIDTracer could be modified to allow more flexibility to the user to match STD trends. For example, an STD CICT tool could potentially allow the user to input several time–step-specific (e.g., 1 week) reproduction numbers to best match the recent trends of reported STD cases. By doing so, the user would provide the tool with a realistic calibration of disease transmission, enabling extrapolations for a certain period. The proportion of under-reported or underdiagnosed cases can be obtained from literature or subject matter experts. Users can perform sensitivity analysis by varying the assumed value and assessing its impact. However, it is possible that the notable differences in STD trends and COVID-19 trends might render the COVIDTracer approach unsuitable for an STD CICT tool. If so, more complex modeling approaches might be required, as discussed in the subsequent section.

### Challenge 3: Key Differences in Mixing Patterns, Anatomic-Specific Transmission, Duration of Infectiousness, and Length of the Incubation Period

**Mixing patterns and anatomic-specific transmission.** The COVIDTracer tool assumes homogeneous mixing,^21^ which means that any given individual is as likely as any other to be infected by some infectious individual.^22^ Usually, acquisition of an STD requires intimate sexual contact, even if briefly, with a person with an STD. This requirement of an intimate connection produces a very different, nonrandom pattern of transmission for STDs from transmission patterns for respiratory diseases.^23^ Mathematical models of STDs have shown the importance of accounting for assortative mixing patterns.^24^ Further modeling complications can also arise for STDs because of anatomic-specific infection. For example, gonococcal infection can occur at exposed anatomical sites such as the urogenital tract, rectum, and pharynx.^25^ Sexual contact must be made with an infected anatomic site to facilitate transmission.^25^^,^^26^ Thus, accounting for site-specific infection and transmission (e.g., urethra-to-rectum, rectum-to-urethra, urethra-to-oropharynx) would add substantial complexity to an STD model,^26^ an issue that is not relevant in COVID-19 models. There may also be heterogeneity in the proportion of sexual contacts who can be named and thus followed up with through contact tracing.^27^

**Duration of infectiousness.** Another difference between COVID-19 and STDs that greatly impacts the ability to produce a simple STD-relevant model of CICT is the difference in duration of infectiousness. With COVID-19, most patients will fully recover and stop shedding the virus in approximately 2 weeks^13^—and perhaps even faster if the patient is treated with antiviral drugs. In contrast, the average duration of infectiousness has been estimated at 361 days for men with chlamydia, 398 days for women with chlamydia, 46 days for men with gonorrhea, and 91 days for women with gonorrhea, in the absence of treatment.^28^ Although treatment reduces the duration of infectiousness, individuals who are asymptomatic may remain untreated,^29^ which increases the duration of infectiousness. Syphilis is most infectious during the primary and secondary stages, which without treatment typically last about 150 days combined; sexual transmission after the first year of infection is relatively rare^24^^,^^30^ (Table 2).

**Table 2:** Selected Estimates of Average Duration of Infectiousness (Range) Through Sexual Transmission in Absence of Treatment and Incubation Period (Range), in Days

Sex	COVID-19	Chlamydia	Gonorrhea	Syphilis
Duration of infectiousness				
Men	11–12	361 (314–423)	46 (39–54)	154
Women	11–12	398 (376–416)	91 (77–109)	154
Incubation period				
Men	4–5	14 (10–18)	6 (4–8)	21 (10–90)
Women	4–5	25 (17–32)	11 (8–15)	21 (10–90)

*Notes:* For COVID-19, differences by sex in the duration of infectiousness and incubation period are not documented; references for these values are provided in Table 1. For chlamydia and gonorrhea, the ranges in parenthesis represent IQRs.^28^ For the incubation period of primary syphilis, they are typically interpreted as the minimum and maximum.^30^ The duration of infectiousness of 154 days for syphilis was calculated as the sum of the mean stay in the primary stage and the mean stay in the secondary stage (46 and 108 days, respectively^24^). No range was provided by Garnett and Anderson,^24^ but sexual transmission after the first year of infection is relatively rare.^24^^,^^30^

**Length of the incubation period.** Although SARS-CoV-2 has an average incubation period of 5 days,^13^^,^^14^ the incubation period is typically longer for gonorrhea (6 days in men, 11 days in women), chlamydia (14 days in men, 25 days in women), and primary syphilis (21 days for men and women)^24^^,^^28^^,^^30^^,^^31^ (Table 2). Given the longer incubation periods and longer duration of infectiousness for these STDs than for COVID-19, assessments of the benefits of CICT for STDs might require a longer time horizon of analysis and might yield a wider range of results in the sensitivity analyses.

#### Addressing Challenge 3

Tools to estimate the impact of CICT on STDs may need to use a more complex disease transmission model.^32^ However, it is possible that the STD CICT tool could incorporate a range of output from a complex model without having to incorporate the model itself. For example, for a given STD, the tool might incorporate information from an external model regarding the estimated number of downstream STDs averted for each infected partner identified and treated through CICT, across a range of scenarios regarding incidence (e.g., low, high), recent trends in incidence (e.g., increasing, decreasing), and other factors. Furthermore, each STD (e.g., chlamydia, gonorrhea, and syphilis) would require its own version of a model.

## DISCUSSION

Recent studies have shown that relatively simple, interactive tools can be used to estimate the impact of CICT on COVID-19.^3^^,^^4^ The tool used for these studies, COVIDTracer, breaks down complex epidemiologic modeling in succinct steps to help public health officials rapidly conduct what would have been otherwise a complicated mathematical analysis. As highlighted in this paper and previous studies,^3^^,^^4^ several factors such as community receptivity and program logistics (e.g., the speed at which contacts are notified) affect the performance and impact of CICT, and the experiences vary greatly across jurisdictions. These factors may depend on financial and logistical barriers^33^ or public acceptance and engagement.^34^ Understanding each jurisdiction's experience and key factors driving the impact of CICT can inform policymakers in designing and improving the program and help to better understand the cost-effectiveness. As such, tools to assess the impact of CICT can be useful not only for COVID-19 but also for other infectious diseases, including STDs such as syphilis, gonorrhea, and chlamydia. However, the suitability of existing tools (STIC Figure and SPACE Monkey) to estimate the impact of CICT on STDs is limited. Given that the STIC Figure and SPACE Monkey tools have proven useful to STD program personnel and other researchers in estimating the impact of STD prevention activities,^35^, ^36^, ^37^ it is likely that a tool that allows more extensive analysis of the benefits of STD CICT would prove useful as well. Although we acknowledge that STDs and COVID-19 are very different in many aspects, our goal is to highlight the benefits of having a simple interactive tool for public health practitioners so that they can readily assess the impact of their STD CICT programs. We also acknowledge that there is a necessity of having a more sophisticated disease transmission model in such STD tools; however, we believe that the benefits of having a user-friendly interface would likely justify the efforts required for development.

An STD tool could benefit from the incorporation of key features from COVIDTracer. First, the STD CICT tool could have an interactive, easy-to-use format to enable public health practitioners to assess the impact of their partner notification activities. Because users of one model (e.g., COVIDTracer) might be likely to use another model (e.g., syphilis, gonorrhea), having some consistency in the user interface would make the models easier to use. Second, the tool could be prepopulated by health departments’ epidemiologists with key data points, so that users would not have to provide an overwhelming amount of local data before using the tool. Third, the tool could be flexible to allow a high degree of customization by users who do have extensive local data and who do wish to include these data in the model. Fourth, the STD CICT tool could include robust sensitivity analyses to illustrate the effects of parameter uncertainty (including those prepopulated by health departments) or parameters that are absent from the literature, model stochasticity (if applicable), key simplifying assumptions, and other factors.

## CONCLUSIONS

The main challenges to the development of an STD CICT tool include the uncertainty around key epidemiologic parameters and the complexity of disease transmission dynamics. Rather than seeing these as limitations preventing the development of such a tool, we argue instead that modeling approaches could still provide reasonable estimates and ranges of the potential impact of CICT activities for STDs. Furthermore, the sensitivity analyses employed by such a model could help in determining what information is needed to improve the effectiveness of the CICT program and the accuracy of the tool.

## CRediT authorship contribution statement

**François M. Castonguay:** Conceptualization, Visualization, Writing – original draft, Validation, Writing – review & editing. **Harrell W. Chesson:** Conceptualization, Writing – original draft, Validation, Writing – review & editing. **Seonghye Jeon:** Conceptualization, Visualization, Validation, Writing – review & editing. **Gabriel Rainisch:** Conceptualization, Visualization, Validation, Writing – review & editing. **Leah S. Fischer:** Conceptualization, Writing – review & editing. **Biswha B. Adhikari:** Conceptualization, Writing – review & editing. **Emily B. Kahn:** Conceptualization, Writing – review & editing. **Bradford Greening:** Conceptualization, Data curation, Methodology, Software. **Thomas L. Gift:** Conceptualization, Validation, Writing – review & editing, Supervision. **Martin I. Meltzer:** Conceptualization, Project administration, Writing – review & editing, Validation, Supervision.
